# Pregnancy-Related Vascular Outcomes in Loeys–Dietz Syndrome: A Retrospective Cohort Study and Case Series

**DOI:** 10.3390/medsci14010079

**Published:** 2026-02-11

**Authors:** Amal Youssef, Hend Bcharah, Hussein Abdul Nabi, George Bcharah, Luke Dreher, Mohammed Alaa Raslan, Fares Jamal, Linnea Baudhuin, Mayowa A. Osundiji, Yuxiang Wang, Christine Firth, Fadi Shamoun

**Affiliations:** 1 Department of Cardiovascular Diseases, Mayo Clinic, Phoenix, AZ 85054, USA; youssef.amal@mayo.edu (A.Y.); osundiji.mayowa@mayo.edu (M.A.O.);; 2 Department of Laboratory Medicine and Pathology, Mayo Clinic, Rochester, MN 55905, USA; 3 Department of Clinical Genomics, Mayo Clinic, Rochester, MN 55905, USA; 4 Department of Clinical Genomics, Mayo Clinic, Pheonix, AZ 85054, USA

**Keywords:** Loeys–Dietz Syndrome, pregnancy outcomes, cardiovascular outcomes, aneurysms, dissections, cardio-obstetrics

## Abstract

**Background:** Loeys–Dietz syndrome (LDS) is an autosomal dominant aortopathy characterized by aggressive aneurysm formation and arterial dissections. Pregnancy-related outcomes and timing of LDS diagnosis remain poorly characterized. **Methods:** Demographics, genetic, obstetric, and vascular data was collected from genetically or clinically confirmed individuals with LDS seen at the three Mayo Clinic sites from 2018 to 2025. Aneurysm progression, new aneurysm formation, and arterial dissections were recorded across all vascular beds. Vascular events were assessed during pregnancy, within 12 months postpartum, and during breastfeeding. Comparative analyses were performed between women with and without a history of pregnancy, and a single-arm descriptive analysis was conducted among patients who experienced vascular complications during the peripartum period. Continuous variables were compared using the Mann–Whitney U test, while categorical variables were analyzed using chi-square or Fisher exact tests. **Results:** Of 47 women with LDS, 24 had a history of pregnancy, accounting for 54 pregnancies. In the comparative analysis, age at LDS diagnosis differed significantly between women with and without a prior pregnancy: women without prior pregnancy were diagnosed at a younger age (median 23.5 years [IQR 10.8–41.0], *n* = 23) than those who had been pregnant (median 53.5 years [IQR 43.0–59.3], *n* = 24). Among pregnant women, the median age at first pregnancy was 28 years (IQR 23–34); only 4 (16.7%) knew their diagnosis before pregnancy. Of 54 pregnancies, 40 (74.1%) resulted in live birth, with 23 (57.5%) vaginal and 17 (42.5%) cesarean deliveries; preterm delivery occurred in 1 (2.5%) pregnancy, and postpartum hemorrhage in 2 (5.0%). No maternal deaths, aortic dissections, or uterine ruptures occurred during gestation or the first postpartum year. In addition, 14 women (58.3%) developed aneurysms, 6 (25.0%) experienced at least one arterial dissection, and 7 (29.2%) required surgical repair, predominantly involving the ascending and abdominal aorta. The prevalence of vascular complications did not differ significantly between groups. **Conclusions:** In this LDS cohort, pregnancy and the early postpartum period were not accompanied by acute aortic catastrophes, despite frequent diagnostic delay. Although women without prior pregnancy were diagnosed at a younger age, the overall burden of vascular and morphologic complications did not differ significantly by pregnancy history. These findings highlight the importance of long-term cardiovascular follow-up in women with LDS.

## 1. Introduction

Loeys–Dietz syndrome (LDS) is an autosomal dominant connective tissue disorder characterized by vascular fragility, aneurysm formation, and dissections across multiple vascular beds [1]. Genetic variants in the transforming growth factor beta (TGF-β) pathway, specifically *TGFBR1*, *TGFBR2*, *SMAD3*, *TGFB2*, *TGFB3*, and *SMAD2*, predispose affected individuals to early-onset aortic aneurysms, dissections, and arterial tortuosity, even at smaller aortic root diameters [1,2,3]. These vascular manifestations are associated with substantial long-term morbidity and premature mortality, driven primarily by aortic and extra-aortic arterial dissections occurring throughout the lifespan [2]. Compared to other connective tissue disorders, including Marfan syndrome and vascular Ehlers–Danlos syndrome, LDS often follows a more aggressive vascular course [2,4].

Pregnancy introduces hormonal and hemodynamic shifts that may exacerbate aortic risk in individuals with LDS [5]. However, data on the timing of diagnosis relative to pregnancy and the nature of vascular complications that occur after delivery or during breastfeeding remain limited. Early reports of LDS in pregnancy, which are largely limited to case studies and small series, suggested high rates of maternal complications during late pregnancy and the postpartum period, including aortic dissection and uterine rupture [5,6,7]. These initial observations raised concerns that women with LDS are at high risk of life-threatening events and potentially fatal vascular events during pregnancy and in the postpartum period [8]. However, more recent studies are redefining the risk of pregnancy in patients with LDS. A multicenter UK study by Cauldwell et al. (2019) reported 20 pregnancies in 13 women with LDS and observed no aortic dissections during pregnancy or postpartum [9]. However, there was a high incidence of obstetric interventions (78% elective cesarean delivery), preterm delivery, postpartum hemorrhage, and aortic root enlargement [9]. Another recent systematic review of 522 LDS pregnancies calculated an overall aortic dissection rate of about 4% and a maternal mortality rate of 1% in the peripartum period [1]. These emerging data suggest that while catastrophic events during pregnancy are becoming more infrequent than initially feared, LDS remains a condition with meaningful long-term vascular risk and non-negligible mortality extending beyond the peripartum period.

Given the limited evidence, this study aims to characterize pregnancy-related vascular outcomes in women with LDS and to describe 5 patients who experienced adverse peripartum events or elected to pursue pregnancy despite clinical recommendations. The purpose of this study is to inform risk stratification, counseling, and management strategies for individuals with LDS who consider or undertake pregnancy.

## 2. Materials and Methods

### 2.1. Study Design and Population

We conducted a multicenter retrospective cohort study across three Mayo Clinic sites (Arizona, Florida, and Minnesota) from 2018 to 2025. Eligible participants were women with a genetically or clinically confirmed diagnosis of LDS; patients with co-existing or overlapping diagnoses of other heritable connective tissue disorders, including Marfan syndrome or Ehlers–Danlos syndrome, were excluded. Clinical data were collected from electronic health records and compiled into a structured dataset. Genetic confirmation required a pathogenic or likely pathogenic variant in an LDS-associated gene. When genetic testing was unavailable or negative, diagnosis was based on documented clinical criteria consistent with LDS. We compared women who had at least one completed pregnancy to women without a pregnancy history. Patients with insufficient documentation were excluded. The final cohort comprised 47 females, 24 of them accounted for 54 pregnancies.

In addition to the overall cohort, we identified a focused case series of five participants who met predefined criteria for clinically significant peripartum risk. The predefined criteria included experiencing a vascular complication (specified below) during pregnancy or within 12 months postpartum or having a known diagnosis of LDS prior to conception while electing to pursue pregnancy despite documented medical recommendations against pregnancy. These five cases were selected to illustrate high-risk clinical scenarios and to provide detailed insight into management challenges and adverse outcomes in LDS.

### 2.2. Data Collection

Clinical data were obtained from detailed chart review of electronic health records, including obstetric notes, cardiovascular imaging reports, operative reports, and genetic testing results. Maternal demographic information (age, race), genotype [pathogenic/likely pathogenic (P/LP) genetic variant, if known], and relevant comorbidities were collected. For each pregnancy, maternal age at conception, gravidity and parity, and whether the diagnosis of LDS was established before or after pregnancy were recorded. Pregnancy outcomes were noted, including live birth, miscarriage or termination, mode of delivery (vaginal vs. cesarean), gestational age at delivery (to identify preterm births), and obstetric complications such as significant postpartum hemorrhage or uterine rupture. When documented, breastfeeding after each pregnancy was recorded.

Vascular complications recorded included all aortic and arterial complications over the course of follow-up. These complications included new aortic dissections, new aneurysm detections, or growth of known aneurysms, as well as any arterial dissections in other territories (e.g., coronary, carotid, visceral arteries). We documented the location and timing of each vascular event and any surgical or endovascular interventions performed. For temporal classification, events were categorized as occurring during pregnancy, within 12 months postpartum, or remote from pregnancy (>12 months postpartum). For each patient, all available aortic imaging (echocardiography, CT/MR angiography) reports were reviewed to track aortic dimensions longitudinally. Imaging surveillance was not protocolized by pregnancy status and was guided by clinical indication rather than standardized intervals. We defined aneurysm progression as an increase in aneurysm size or the formation of a new aneurysm compared to baseline, and disease progression as any combination of aneurysm growth, new aneurysm formation, or arterial dissection occurring over the follow-up period.

### 2.3. Outcomes

The primary outcome was the occurrence of a vascular complication (aortic or extra-aortic dissection, new/progressive aneurysm) during pregnancy, within 12 months postpartum, or during breastfeeding. Secondary outcomes included timing of LDS diagnosis relative to pregnancy, maternal obstetric complications, and fetal/neonatal outcomes (live birth, preterm delivery, loss). Comparative analyses focused on differences in vascular and clinical characteristics between women with and without a history of pregnancy. Results were summarized per patient and per pregnancy to account for multiple pregnancies in individual women.

### 2.4. Statistical Analysis

Data were analyzed descriptively. Continuous variables are reported as medians with interquartile ranges (IQR) and compared using the Mann–Whitney U test. Categorical variables were expressed as frequencies and percentages and analyzed using Chi-square or Fisher exact tests, as appropriate. All tests were two-sided, and exact *p*-values are reported where applicable, with statistical significance defined as a *p*-value < 0.05. Given the descriptive and exploratory nature of the study, no formal adjustment for multiple comparisons was performed. Analyses were performed using IBM SPSS 29.0.0.

## 3. Results

### 3.1. Cohort Characteristics

**Figure 1:**
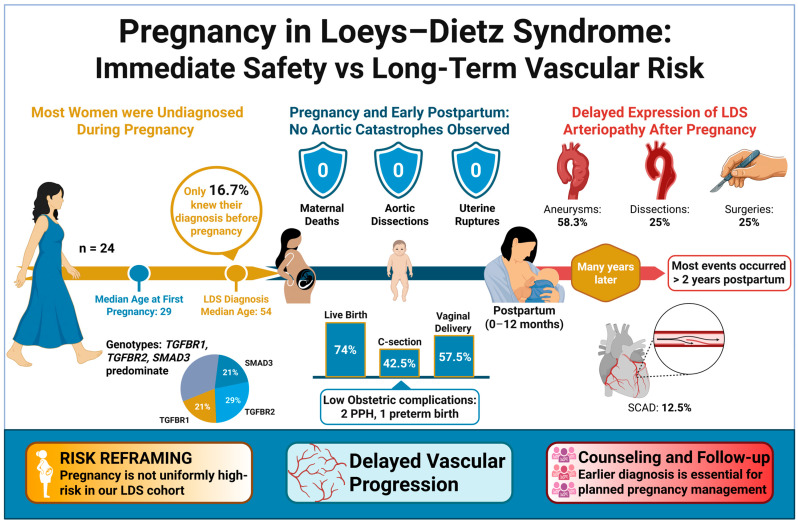
Immediate peripartum outcomes and delayed vascular manifestations in women with LDS: The schematic depicts pregnancy-related and long-term vascular outcomes in women with LDS and a history of pregnancy (*n* = 24; 54 pregnancies). Most women were diagnosed with LDS after completion of childbearing. No acute aortic catastrophes occurred during pregnancy or within 12 months postpartum. However, aneurysm formation, arterial dissections (including spontaneous coronary artery dissection) and surgical interventions were frequently identified during long-term follow-up, with most events occurring more than two years postpartum. The genotype distribution of affected women is shown, highlighting heterogeneity in vascular expression over time. “Created in BioRender. Dreher, L. (2026) https://BioRender.com/yxlks00”.

Among 47 women who were diagnosed with LDS, 24 had a collective history of 54 pregnancies. Most women were diagnosed with LDS after completion of childbearing, reflecting delayed clinical recognition rather than absence of pregnancy-related risk. Race distribution, gene grouping, and mutation type did not differ significantly between groups. Across all assessed cardiovascular outcomes including arterial dissections, aneurysms across multiple vascular beds, prior vascular or valvular surgery, and emergent interventions, no statistically significant differences were observed between pregnant and non-pregnant women. These data are summarized in Appendix A Table A1. Figure 1 provides an overview of the key results and outcomes of the study.

**Figure 2:**
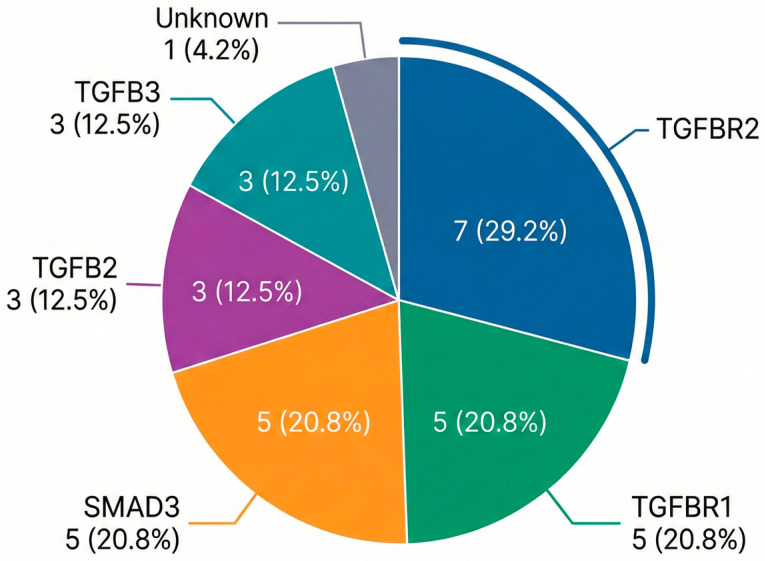
Genotype Distribution of Patients with LDS with a history of pregnancy: Pie chart showing the proportion and absolute number of individuals by affected gene, including *TGFBR2*, *TGFBR1*, *SMAD3*, *TGFB2*, *TGFB3*, and cases with unknown genotypes. Values are presented as N(%) of the total cohort.

In a subgroup analysis of patients with a prior history of pregnancy, the median age at the first pregnancy was 29 years (range 23–34 years), with a median age at formal LDS diagnosis of 54 years (43–59). No maternal deaths occurred in the cohort. Baseline patient characteristics were detailed in Table 1. The most prevalent P/LP variants in this cohort were TGFBR2 (29.2%), TGFBR1 (20.8%), and SMAD3 (20.8%). The genotype distribution for the cohort is shown in Figure 2. Information on family screening, inheritance patterns in offspring, and fetal genetic outcomes was inconsistently available due to the retrospective nature of the study and reliance on external obstetric and genetic records; therefore, these data could not be systematically analyzed.

### 3.2. Obstetric and Pregnancy Outcomes

Of the 54 total pregnancies, 40 (74.1%) resulted in live birth. There was one documented fetal demise and 13 miscarriages or terminations. No maternal deaths, aortic dissections, or uterine ruptures occurred during pregnancy. Pregnancy and obstetric outcomes are summarized per-pregnancy and per-delivery in Table 2.

### 3.3. Lifetime Vascular Complications

A total of 14 patients (58.3%) had at least one aneurysm; 6 (25.0%) experienced at least one dissection, and 7 (29.2%) required surgical repair throughout their lives. The ascending aorta was the most common site for aneurysms (58.3%), followed by the abdominal aorta (37.5%) and cerebral arteries (29.1%). Arterial dissections were most frequent in the coronary arteries [spontaneous coronary artery dissection (SCAD)], occurring in 3 patients (12.5%). Two patients (8.3%) had an ascending aortic dissection, and one (4.2%) had an abdominal aortic dissection. Notably, most of these vascular events occurred more than two years postpartum. The prevalence of vascular events recorded throughout each patient’s lifetime is presented in Table 3.

### 3.4. Case Series of Five Patients

Five representative cases (Cases 1–5) were selected to illustrate the spectrum of disease and timing of complications relative to pregnancy (Table 4). The timing of LDS diagnosis varied considerably: one case (Case 3) was diagnosed before pregnancy, three cases (1, 4, 5) were diagnosed in the postpartum period, and one case (Case 2) was diagnosed years after pregnancy. Three of the five cases experienced significant vascular or cardiovascular complications in the postpartum period: one postpartum SCAD (spontaneous coronary artery dissection) (Case 1), one recurrent renal artery dissection during pregnancy (Case 3), and one instance of postpartum heart failure secondary to severe aortic regurgitation requiring urgent root replacement (Case 4).

#### 3.4.1. Case 1

A woman with a pathogenic *SMAD3* variant had her first pregnancy at age 38. Five months following an uncomplicated vaginal delivery, she experienced SCAD of the mid-left anterior descending (LAD) artery. This event led to her formal diagnosis of LDS at age 39. She subsequently had a second, uncomplicated vaginal delivery at age 41. Transthoracic echocardiography demonstrated preserved left ventricular systolic function (LVEF 59%) with normal ventricular size, no pulmonary hypertension, and no hemodynamically significant valvular disease; Although her aorta remained stable during both pregnancies, gradual enlargement of 5 mm (37–42 mm) over the seven years after her last delivery prompted a successful valve-sparing aortic root replacement. Postoperative computed tomography angiography confirmed stable graft dimensions (36–37 mm at the root and 22–26 mm in the mid-ascending aorta). She was later diagnosed with aneurysmal dilatation of the ophthalmic artery and fibromuscular dysplasia involving the carotid, vertebral, renal, splenic, and iliac arteries.

#### 3.4.2. Case 2

A woman with pathogenic *TGFBR2* variant was identified during family screening at age 43, six years after her last live birth. Her obstetric history includes two cesarean section deliveries (C-sections) (ages 32 and 37) and one missed abortion (at age 44); both live births were complicated by postpartum hypertension. Her aortic root and ascending aorta have shown slow, progressive dilation, increasing from 33/31 mm to 44/38 mm over 10 years. Transthoracic echocardiography showed preserved LVEF 64% with normal ventricular size, no pulmonary hypertension, and no hemodynamically significant valvular disease. The aortic valve area was within normal limits (2.74 cm^2^). She has not required surgical intervention to date.

#### 3.4.3. Case 3

A woman with a likely pathogenic TGFBR1 variant was diagnosed with LDS at age 28, prior to any pregnancies, after presenting with a left distal main renal artery dissection. She had two pregnancies, both delivered by C-section. Her first pregnancy at age 30 was complicated by a recurrent left renal artery dissection early in gestation, which was managed conservatively. Her second pregnancy at age 32 was uncomplicated. Serial transthoracic echocardiography demonstrated preserved LVEF 55% with normal ventricular size, no pulmonary hypertension, and no hemodynamically significant valvular dysfunction. The aortic valve was bicuspid with a normal valve area (2.77 cm^2^). Aortic dimensions remained stable and within normal limits throughout pregnancy and long-term follow-up (sinus of Valsalva 28–30 mm; mid-ascending aorta 22–31 mm). No progressive aortic dilation or aortic dissection was observed during her obstetric course or subsequent surveillance.

#### 3.4.4. Case 4

A woman with a pathogenic *SMAD3* variant and severe scoliosis had four vaginal deliveries. She was diagnosed with LDS at age 38, five months after her fourth pregnancy, when she presented with acute postpartum heart failure (EF 20–30%) with severe aortic regurgitation. This was attributed to severe aortic root enlargement to 6.0 cm, which had progressed from 4.8 cm pre-pregnancy. She required emergent valve-sparing root replacement, which was complicated by residual regurgitation and cardiogenic shock (LVEF ~20%). She required a redo mechanical aortic valve replacement one year later. She also developed severe multisystem vasculopathy, including multiple visceral (superior mesenteric artery (SMA), hepatic, splenic), iliac, and cerebral aneurysms, as well as chronic SMA dissection, requiring multiple repairs. Serial echocardiography following surgical repair demonstrated stable post-repair aortic dimensions (sinus of Valsalva ~33–34 mm; mid-ascending aorta 29–42 mm across follow-up) with recovery of left ventricular systolic function (LVEF ~65%), no evidence of prosthetic valve dysfunction, and no pulmonary hypertension. She has remained clinically stable on long-term follow-up with no recurrent aortic or visceral dissections since 2019 and continues multidisciplinary surveillance through cardiogenetic, vascular surgery, and longitudinal imaging programs.

#### 3.4.5. Case 5

A woman with a pathogenic *TGFBR1* variant was diagnosed with LDS at age 26, one year after her first pregnancy. Her diagnosis was made by family screening. Her obstetric history consists of one uncomplicated C-section at term, after which she breastfed. Transthoracic echocardiography demonstrated preserved LVEF (55%) with normal ventricular size, no evidence of systolic dysfunction, and no pulmonary hypertension. The aortic valve was bicuspid with a normal valve area (2.77 cm^2^), without hemodynamically significant stenosis, and aortic dimensions were within normal limits (sinus of Valsalva 30 mm, mid-ascending aorta 31 mm). Postpartum imaging later revealed an ascending aortic aneurysm and a small, stable cerebral aneurysm.

## 4. Discussion

This retrospective multicenter cohort describes pregnancy-related and long-term vascular outcomes in 47 women with LDS encompassing 54 pregnancies, reflecting an average of approximately two pregnancies per patient. The median age at LDS diagnosis was 54 years, with most patients diagnosed with LDS well after childbearing age. Despite the lack of LDS-directed management during pregnancy, maternal survival through all 54 pregnancies was 100%, with no maternal deaths during the peripartum period. In addition, no aortic dissection was observed during gestation or in the immediate 12-month postpartum window, despite only 16.7% of patients (4 out of 24) had known LDS diagnosis prior to pregnancy. Consequently, most of the pregnancies proceeded without specialized surveillance that would be recommended for a known aortopathy. These findings describe pregnancy tolerance in this cohort but should not be interpreted as evidence of low intrinsic pregnancy risk in LDS.

However, LDS-related vascular complications tended to occur years after pregnancy. In this cohort, every aneurysm that was noted or eventually treated was discovered years after completion of childbearing. Six patients (25%) experienced at least one arterial dissection in the long-term follow-up [10]. The postpartum period, especially the first 6–12 months after birth, is known to be a vulnerable time for aortic dissection in connective tissue disorders like Marfan syndrome [8,11]. While we did not observe any immediate postpartum aortic dissections in our series, we did see a case of postpartum SCAD (patient 1). In addition, case 4 had significant expansion of the aortic root in the postpartum period. Several patients were found to have new aneurysms or progressive enlargement of existing aneurysms on imaging within a few years after pregnancy. These delayed and heterogeneous vascular manifestations are illustrated in the five-case series summarized in Table 4, which links genotype, timing of LDS diagnosis relative to pregnancy, and subsequent vascular outcomes. This suggests that pregnancy may act as a physiological stressor that accelerates underlying arteriopathies in LDS, with clinical manifestations (aneurysm progression reaching a threshold or arterial wall dissection) becoming evident later in life.

The relatively low rate of acute peripartum vascular complications observed in this cohort should be interpreted in the context of delayed LDS diagnosis and potential survivor bias. These findings likely reflect a combination of under-ascertainment of fatal events and the retrospective nature of diagnosis rather than true absence of pregnancy-associated risk.

Prior studies have shown the delayed effects of pregnancy on vascular growth in patients with connective tissue disease. Women with Marfan syndrome who had been pregnant exhibited faster aortic-root enlargement (~0.64 mm/year vs. 0.12 mm/year in nulligravid women), with the greatest expansion occurring during the first postpartum year [12,13,14]. While LDS is a distinct condition, it shares the common features of *TGF-β* dysregulation and aortic wall structural weakness with Marfan syndrome [15,16]. It is therefore plausible that pregnancy exerts a lasting impact on the aorta in LDS. The hemodynamic load of gestation (~50% increase in blood volume and cardiac output) and the postpartum hormonal milieu (sudden changes in estrogen/progesterone and possibly lingering effects on vessel connective tissue) might trigger or accelerate arterial remodeling [11]. The finding that all significant aneurysms and dissections were postpartum supports this, as pregnancy may act as a stressor on the vascular system, unmasking latent predisposition that then evolves into clinical disease over subsequent years.

The pattern of vascular involvement outside the aorta should also be noted. LDS mainly affects medium-sized arteries in addition to the aorta [17]. Consistent with this, most patients in this cohort had carotid, cerebral, splenic, and mesenteric aneurysms and dissections. Most importantly, SCAD was seen in three patients, which is a common cause of myocardial infarction in young women and is often associated with postpartum status and connective tissue disorders, particularly fibromuscular dysplasia (FMD). In case 1, the combination of LDS and FMD likely predisposed her to develop a SCAD five-month postpartum. These findings underscore the need for standardized comprehensive vascular surveillance that extends beyond the aorta, especially during the postpartum period.

The timing of LDS diagnosis has important implications. The median age of diagnosis in this cohort was 54 years, well past typical childbearing years. Most women (83%) were only diagnosed after an aortic event that occurred later in life, often years after their last pregnancy. This likely affected the findings, especially when compared to previously published series of LDS pregnancies, where most women were diagnosed with LDS prior to their pregnancy. This allowed for interventions like elective cesarean at 37 weeks in majority (78% of deliveries) and aggressive postpartum monitoring [9]. Despite this, the outcomes in terms of aortic dissection were similar (no occurrence during pregnancy and early postpartum in both cohorts). However, our cohort had fewer complications (17% preterm rate and 4% PPH). This difference could be partly due to our retrospective design and differences in management, considering that routine elective early deliveries can increase preterm birth rate and might predispose to cesarean related blood loss, compared with our cohort, in which the majority of women delivered spontaneously at term. Fortunately, the lack of specialized intervention did not result in harm in our cohort; however, we caution that this may not be generalizable to the general LDS population, as this does not eliminate vascular risk. Importantly, comparisons between women with and without a history of pregnancy in this study are descriptive in nature and may be confounded by differences in age and age at diagnosis; therefore, causal inferences regarding the effect of pregnancy on vascular outcomes cannot be made.

As summarized in Table 4, our case series highlights the heterogeneity of LDS manifestations. In our cohort, the individuals who experienced the earliest and most dramatic complications (Cases 1 and 4) had *SMAD3* variants, whereas Case 5 (*TGFBR1*) and Case 2 (*TGFBR2*) had significant aortic aneurysms by their 40s, and Case 3 (*TGFBR1*) had virtually no aortic growth through her 30s. This mirrors LDS genotype-phenotype trends seen in the literature. Prior research has shown that *TGFBR1* or *TGFBR2* (also known as LDS types 1 and 2) variants are often associated with more aggressive disease in terms of early and widespread aneurysms [18]. *SMAD3* variants (LDS type 3) also carry high aortic risk and can cause early-onset osteoarthritis and skeletal findings (aneurysm-osteoarthritis syndrome) [19]. In contrast, variants in *TGFB2* or *TGFB3* (LDS types 4 and 5) tend to have milder phenotypes and late onset aneurysms [1,8]. These genotypic differences emphasize the need for personalized management, and patients with LDS warrant diligent follow-up, especially after pregnancy.

Based on the findings of this study, pregnancies in women with Loeys–Dietz syndrome should be managed as high-risk, even in the absence of a known diagnosis at the time of pregnancy, with early involvement of a multidisciplinary cardio-obstetrics team including maternal–fetal medicine specialists, cardiology, genetics, and anesthesiology. Preconception counseling should be pursued whenever possible and should address maternal vascular risk, inheritance patterns, and the limitations of aortic size alone in predicting dissection, while pregnancy management should include individualized surveillance strategies and careful blood pressure control when LDS is known or suspected. Importantly, the delayed and heterogeneous vascular manifestations observed in this cohort highlight critical gaps in current knowledge and underscore the need for prospective, longitudinal studies within this population to better define pregnancy-associated and long-term vascular risk, optimal imaging intervals, and genotype-specific trajectories. Given the propensity for extra-aortic vascular involvement and postpartum events such as spontaneous coronary artery dissection, future studies should incorporate comprehensive vascular surveillance beyond the aorta and extend follow-up well beyond the traditional postpartum period. Such efforts are essential to inform evidence-based counseling, refine risk stratification, and guide long-term multidisciplinary care for women with LDS.

## 5. Conclusions

In this retrospective cohort, no acute aortic dissections were observed during pregnancy or within the first 12 months postpartum among women with LDS. However, these findings should be interpreted in the context of delayed LDS diagnosis, limited antenatal vascular imaging, and potential survivor bias. The absence of observed acute peripartum aortic catastrophes in this cohort does not equate to absence of pregnancy-related vascular risk. Rather, pregnancy may act as a physiological stressor that contributes to longer-term vascular disease progression, with clinically evident complications emerging years later. These data underscore the importance of cautious counseling, individualized risk assessment, and continued long-term vascular surveillance in women with LDS who have a history of pregnancy.

## 6. Limitations

This study is limited by its retrospective design. All pregnancy-related information was obtained from the electronic health record which sometimes contained fragmented or incomplete external documentation of some outcomes. Therefore, gaps in obstetric histories and limited standardized data capture across all pregnancies were present. As a result, some outcomes may be underreported or inconsistently documented.

Many patients in our cohort were diagnosed with LDS later in life, after their childbearing years. This delayed diagnosis limits availability of vascular imaging during pregnancy and introduces potential survivor bias, as individuals who may have experienced catastrophic pregnancy-related vascular events could be underrepresented. Additionally, variable follow-up duration and non-standardized imaging intervals limited more granular temporal stratification of vascular events beyond the defined pregnancy and postpartum periods.

## Figures and Tables

**Table 1:** Baseline characteristics of pregnant patients with a history of pregnancy.

Characteristics	Value
Age at first pregnancy, y	Median (IQR): 29 (23–34)
Age at LDS * diagnosis, y	Median (IQR): 54 (43–59)
Race, White	23 (95.8%)
Family history of vascular complications	Positive 18 (75.0%) Negative 4 (16.7%) Unknown 2 (8.3%)
Death	0
Comorbidities	
Obesity	3 (12.5%)
Hypertension	4 (16.7%)
DM *	0 (0%)
Hyperlipidemia	1 (4.2%)
Migraine	4 (16.7%)
Thyroid Dx	2 (8.3%)

* LDS: Loeys–Dietz syndrome; DM: Diabetes mellitus.

**Table 2:** Pregnancy, Obstetric, and Postpartum Outcomes (*N* = 24 Patients; *N* = 54 Pregnancies).

Outcome	Value
**Pregnancy Outcomes**	Total *N* = 54 Pregnancies
Live Birth	40/54 (74.1%)
Fetal Demise	1/54 (1.9%)
Miscarriage, Termination, or Unknown	13/54 (24.0%)
**Delivery Characteristics**	Total *N* = 40 Live Births
Vaginal Delivery	23/40 (57.5%)
Cesarean Delivery	17/40 (42.5%)
Preterm Delivery (<37 weeks)	1/40 (2.5%)
**Maternal Obstetric Complications**	Total *N* = 40 Live Births
Postpartum Hemorrhage (>1000 mL)	2/40 (5.0%)
Uterine Rupture	0/40 (0%)
Pregnancies followed by breastfeeding	11/40 (27.5%)

**Table 3:** Prevalence of Lifetime Vascular Events by Location (*N* = 24).

Vascular Site	Aneurysm, *N* (%)	Dissections, *N* (%)	Repairs, *N* (%)
Total patients affected	14 (58.3%)	6 (25.0%)	7 (29.2%)
Abdominal aorta	9 (37.5%)	1 (4.2%)	7 (29.2%)
Ascending aorta	14 (58.3%)	2 (8.3%)	7 (29.2%)
Carotid artery	4 (16.7%)	0	0
Cerebral artery	7 (29.1%)	0	0
Coronary artery	1 (4.2%)	3 (12.5%)	1 (4.2%)
Mesenteric artery	2 (8.3%)	1 (4.2%)	0
Celiac artery	1 (4.2%)	0	0
Splenic artery	3 (12.5%)	1 (4.2%)	0
Iliac artery	2 (8.3%)	1 (4.2%)	1 (4.2%)
Renal artery	1 (4.2%)	1 (4.2%)	0
Vertebral artery	0	1 (4.2%)	0

**Table 4:** Key clinical, genetic, and pregnancy-related features of five illustrative Loeys–Dietz syndrome cases in relation to cohort-level vascular outcomes.

Feature	Case 1	Case 2	Case 3	Case 4	Case 5
**Gene**	*SMAD3*	*TGFBR2*	*TGFBR1*	*SMAD3*	*TGFBR1*
**Variant**	*c.872-1G>C*	*c.1378C>T*	*c.844T>C*	*c.455delC*	*c.1164_1176delinsCG*
**Timing of LDS diagnosis relative to pregnancy**	Postpartum	Remote postpartum	Before pregnancy	Postpartum	Remote postpartum
**Family history**	Positive	Positive	Negative	Positive	Positive
**Systemic symptoms**	Joint hypermobility; FMD *	Postpartum HTN *	Recurrent DVT *	Severe scoliosis; A-fib *	none
**Aortic disease status**	Progressive	Progressive	Stable	Progressive	Stable
**Cardiac function**	Normal EF *	Normal EF	Normal HF	Postpartum HF *	Normal EF
**Cardiovascular medications**	metoprolol	Losartan	Lisinopril, ASA *, apixaban	Losartan + BB * + AC *	none
**Timing and type of vascular or obstetric complication relative to pregnancy**	Postpartum SCAD *	Postpartum HTN	Renal artery dissection	Post partum HF and AR * requiring replacement	none
**Breastfeeding**	Positive	Positive	Positive	Positive	Positive
**Interventions**	Valve-sparing root replacement	none	none	Valve-sparing root replacement, mechanical AVR *, multiple aneurysm repairs	none

* FMD: fibromuscular dysplasia; HTN: hypertension; DVT: deep vein thrombosis; A-fib: atrial fibrillation; EF: Ejection fraction; ASA: acetylsalicylic acid; HF: heart failure; BB: β-blocker; AC: Anticoagulant.

## Data Availability

The data supporting the findings of this study are available from the corresponding author upon reasonable request. Due to institutional regulations and patient confidentiality agreements, the raw data is not publicly available.

## Abbreviations

The following abbreviations are used in this manuscript:

AR	Aortic regurgitation
AVR	Aortic valve replacement
EF	Ejection fraction
FMD	Fibromuscular dysplasia
HF	Heart failure
SCAD	Spontaneous coronary artery dissection

## Appendix A

**Table A1:** Demographic, genetic, and cardiovascular characteristics of women with LDS stratified by pregnancy history.

	Pregnant *N* = 24	Non-Pregnant *N* = 23	Total *N* = 47	*p*-Value
Current age	57 [44.8–67.3]	28 [19.5–47.5]	47 (100%)	0.0000327
Age at diagnosis	48.5 [41–57.75]	21 [8–44.5]	47 (100%)	0.000016
Race, white	23 (95.8%)	20 (86.9%)	47 (100%)	0.34
Death	0	2 (8.7%)	47 (100%)	0.23
**Gene Grouping**			47 (100%)	0.64
*TGFBR1*	5 (20.8%)	8 (34.8%)		
*TGFBR2*	7 (29.2%)	7 (30.4%)		
*SMAD3*	4 (16.7%)	2 (8.7%)		
*TGFB2*	2 (8.3%)	2 (8.7%)		
*TGFB3*	2 (8.3%)	0		
**Type of Mutation**			47 (100%)	0.56
Deletion	1 (4.1%)	1 (4.3%)		
Deletion and Insertion	3 (12.5%)	2 (8.7%)		
Duplication	1 (4.1%)	2 (8.7%)		
Missense	13 (54.2%)	15 (65.2%)		
Nonsense	3 (12.5%)	0		
Splice site	1 (4.1%)	0		
**Dissections**				
Vertebral artery	3 (12.5%)	1 (4.3%)	47 (100%)	0.61
Carotid artery	1 (4.1%)	1 (4.3%)	47 (100%)	0.9
Renal artery	1 (4.1%)	0	47 (100%)	0.9
Coronary artery	1 (4.1%)	0	47 (100%)	0.9
Innominate artery	0	1 (4.3%)	47 (100%)	0.48
Type A dissection	1 (4.1%)	2 (8.7%)	47 (100%)	0.61
Type B dissection	1 (4.1%)	2 (8.7%)	47 (100%)	0.61
Vertebral artery	3 (12.5%)	1 (4.3%)	47 (100%)	0.61
**Aneurysms**				
Carotid artery	2 (8.3%)	2 (8.7%)	47 (100%)	0.9
Vertebral artery	1 (4.1%)	0	47 (100%)	0.9
Splenic artery	3 (12.5%)	2 (8.7%)	47 (100%)	0.9
Mesenteric or celiac	4 (16.7%)	3 (13%)	47 (100%)	0.9
Renal artery	2 (8.3%)	0	47 (100%)	0.48
Iliac and femoral artery	3 (12.5%)	3 (13%)	47 (100%)	0.9
Subclavian artery	0	2 (8.7%)	47 (100%)	0.23
Cerebral artery	6 (25%)	5 (21.7%)	47 (100%)	0.9
Coronary artery	2 (8.3%)	0	47 (100%)	0.48
Ascending aorta	15 (62.5%)	19 (82.6%)	47 (100%)	0.19
Abdominal aorta	2 (8.3%)	3 (13%)	47 (100%)	0.66
Carotid artery	2 (8.3%)	2 (8.7%)	47 (100%)	0.9
Vertebral artery	1 (4.1%)	0	47 (100%)	0.9
**Surgeries**				
Hernia repair	10 (41.7%)	5 (21.7%)	47 (100%)	0.21
Dissection or aneurysm repair	12 (50%)	17 (73.9%)	47 (100%)	0.13
Emergent due to dissection	1 (4.1%)	1 (4.3%)	47 (100%)	0.9
MVR *	2 (8.3%)	5 (21.7%)	47 (100%)	0.24
TVR *	4 (16.7%)	5 (21.7%)	47 (100%)	0.72
AVR *	8 (33.4%)	8 (34.8%)	47 (100%)	0.9

* MVR: mitral valve replacement, TVR: tricuspid valve replacement, AVR: Aortic valve replacement.
